# A Bayesian network model of new-onset diabetes in older Chinese: The Guangzhou biobank cohort study

**DOI:** 10.3389/fendo.2022.916851

**Published:** 2022-08-03

**Authors:** Ying Wang, Wei Sen Zhang, Yuan Tao Hao, Chao Qiang Jiang, Ya Li Jin, Kar Keung Cheng, Tai Hing Lam, Lin Xu

**Affiliations:** ^1^ School of Public Health, Sun Yat-Sen University, Guangzhou, China; ^2^ Molecular Epidemiology Research Centre, Guangzhou Twelfth People’s Hospital, Guangzhou, China; ^3^ Institute of Applied Health Research, University of Birmingham, Birmingham, United Kingdom; ^4^ School of Public Health, The University of Hong Kong, Hong Kong SAR, China

**Keywords:** Bayesian network, directed acyclic graph, causal model, risk factors, diabetes

## Abstract

**Background:**

Existing diabetes risk prediction models based on regression were limited in dealing with collinearity and complex interactions. Bayesian network (BN) model that considers interactions may provide additional information to predict risk and infer causation.

**Methods:**

BN model was constructed for new-onset diabetes using prospective data of 15,934 participants without diabetes at baseline [73% women; mean (standard deviation) age = 61.0 (6.9) years]. Participants were randomly assigned to a training (n = 12,748) set and a validation (n = 3,186) set. Model performances were assessed using area under the receiver operating characteristic curve (AUC).

**Results:**

During an average follow-up of 4.1 (interquartile range = 3.3–4.5) years, 1,302 (8.17%) participants developed diabetes. The constructed BN model showed the associations (direct, indirect, or no) among 24 risk factors, and only hypertension, impaired fasting glucose (IFG; fasting glucose of 5.6–6.9 mmol/L), and greater waist circumference (WC) were directly associated with new-onset diabetes. The risk prediction model showed that the post-test probability of developing diabetes in participants with hypertension, IFG, and greater WC was 27.5%, with AUC of 0.746 [95% confidence interval CI) = 0.732–0.760], sensitivity of 0.727 (95% CI = 0.703–0.752), and specificity of 0.660 (95% CI = 0.652–0.667). This prediction model appeared to perform better than a logistic regression model using the same three predictors (AUC = 0.734, 95% CI = 0.703–0.764, sensitivity = 0.604, and specificity = 0.745).

**Conclusions:**

We have first reported a BN model in predicting new-onset diabetes with the smallest number of factors among existing models in the literature. BN yielded a more comprehensive figure showing graphically the inter-relations for multiple factors with diabetes than existing regression models.

## Introduction

Diabetes mellitus is a major health problem worldwide (1). According to the International Diabetes Federation, 463 million (9.3%) adults lived with and 4.2 million died from diabetes in 2019. The number of diabetic patients would increase to 700 million by 2045 if the current trend continues (2, 3). Diabetes causes premature mortality, serious complications, and lower quality of life (1), with huge economic burden on countries and health systems for individuals and their families. An accurate prediction tool for risk stratification may enable identification of high-risk individuals for early prevention and intervention (4).

Previous diabetes prediction models including ours (5) from traditional regression–based techniques were limited in dealing with collinearity and complex interactions (3, 5, 6). As diabetes is a multifactorial disease and some risk factors are correlated, distinguishing causal from non-causal correlation factors can enable more targeted and effective interventions (3). Although Mendelian randomization (MR) is widely used to identify likely causal associations (7), it fails to account for interaction among multiple factors and relies on the existence of validated genetic instrumental variables.

Alternatively, directed acyclic graph (DAG) is a graphical tool that provides a simple way to visually represent and better understand the key concepts of exposure, outcome, mediation, and confounding (8). Bayesian network (BN) is based on DAG, which has been used for clinical prediction, disease diagnosis, and causal exploration (9). BN model has advantages of identifying the interactions among exposures, describing direct and indirect associations between exposures and disease and outputting an intuitive conditional probability table for decision-making. It is useful for knowledge representation and effective inference under uncertainty (10). Despite its increasing application in medical research (11), we searched PubMed using keywords of “diabetes” and (“Bayesian network” or “Bayes network” or “belief network” or “causal network” or “directed acyclic graphs”) up to March 2022 and found no population-based cohort studies using BN model in predicting new-onset diabetes risk and identifying network associations of the risk factors.

We hence used BN to integrate interactions among multiple exposures, identify likely causal associations of a large number of possible risk factors, and construct a risk prediction model for new-onset diabetes using data from the Guangzhou Biobank Cohort Study (GBCS). We aimed to visually display a novel framework of risk prediction and the likely casual associations to aid better understanding of disease mechanisms and risk stratification.

## Materials and methods

The baseline examination of GBCS was done during September 2003 to January 2008. The present study included 18,105 participants who returned for follow-up examination during March 2008 to December 2012. Details of the GBCS have been reported elsewhere (5, 12). Briefly, GBCS is a three-way collaborative project among the Guangzhou Twelfth People’s Hospital and the Universities of Hong Kong and Birmingham. Participants were recruited from the Guangzhou Health and Happiness Association for the Respectable Elders, which is a community social and welfare organization with branches in all districts of Guangzhou, Guangdong Province. A total of 7% of local residents aged 50+ years enrolled in the GHHARE with a nominal membership fee of 4 RMB (≈50 US cents) per month.

The baseline and follow-up examinations were conducted by well-trained nurses and technicians. Information of demographic characteristics, lifestyle factors, and family and personal medical history was collected by a computer-based questionnaire in face-to-face interview, and assessments of anthropometrics (i.e., weight, height, waist, and hip circumference), blood pressure, fasting plasma glucose (FPG), lipids, and inflammatory markers were done using a standard protocol. Fasting blood samples were drawn using a Vacutainer tube. Fasting plasma lipids and glucose were measured in the hospital laboratory. After an initial 5-min rest, seated blood pressure was measured three times at 1-min intervals using Omron 705CP sphygmomanometer (Omron Corp, Kyoto, Japan). Weight, standing height, sitting height, waist circumference (WC), and hip circumference were measured with light indoor clothing and without shoes. Details of the variable measurements have been reported elsewhere (12). Impaired fasting glucose (IFG) was defined as FPG levels of ≥ 5.6 mmol/L and ≤ 6.9 mmol/L (13). GBCS was approved by the Guangzhou Medical Ethics Committee of the Chinese Medical Association. All participants signed the informed consent form before participation.

### Definition of diabetes

Diabetes was defined by FPG ≥7.0 mmol/L, self-reported physician-diagnosed diabetes, or use of hypoglycemia medication or insulin at baseline. Few participants reported type 1 diabetes (n = 73) at baseline, and they were excluded. New-onset type 2 diabetes (shortened as diabetes) was defined by FPG ≥7.0 mmol/L at the follow-up examination, with self-reported physician-diagnosed diabetes, or initiation of hypoglycemic medications or insulin during the follow-up period (14).

### Bayesian network

We used BN to integrate interactions among multiple exposures and construct a risk prediction model for new-onset diabetes (15). BN is a graphic model whose structure consists of a series of nodes (variables) connected by arcs in a directed fashion. All nodes in a BN represent attributes or variables of the model, with conditional probability used to represent the probability of each possible event in a child node and given each possible event in the parent node. The BN model is composed of two tuples presenting as B = (Bs, Bp), where Bs represents the DAG, and Bp represents a set of parameters that quantify the graph edges by specifying the conditional probability distributions; in the discrete case, they are represented as conditional probability tables. The arcs in DAG represent conditional dependence relationships (in most cases, a causal link) between variables. The value of each node in the model depends on the parent node (16). The probability distribution of a node can be expressed by the product of probability of each node, as follows:


$$\text{P}\left(X_{1},\ldots ,X_{n}\right)=\prod_{i=1}^{n}P(X_{i}|Pa\left(X_{i}\right))$$

Here, *Pa*(*X_i_*) BN learning consists of two tasks: structural learning and parametric learning. Structure learning obtains the network topology structure and combines the training data and empirical expertise. Parameter learning can obtain the conditional probability distributions by analyzing the conditional dependence relationship between nodes.

**Figure 1:**
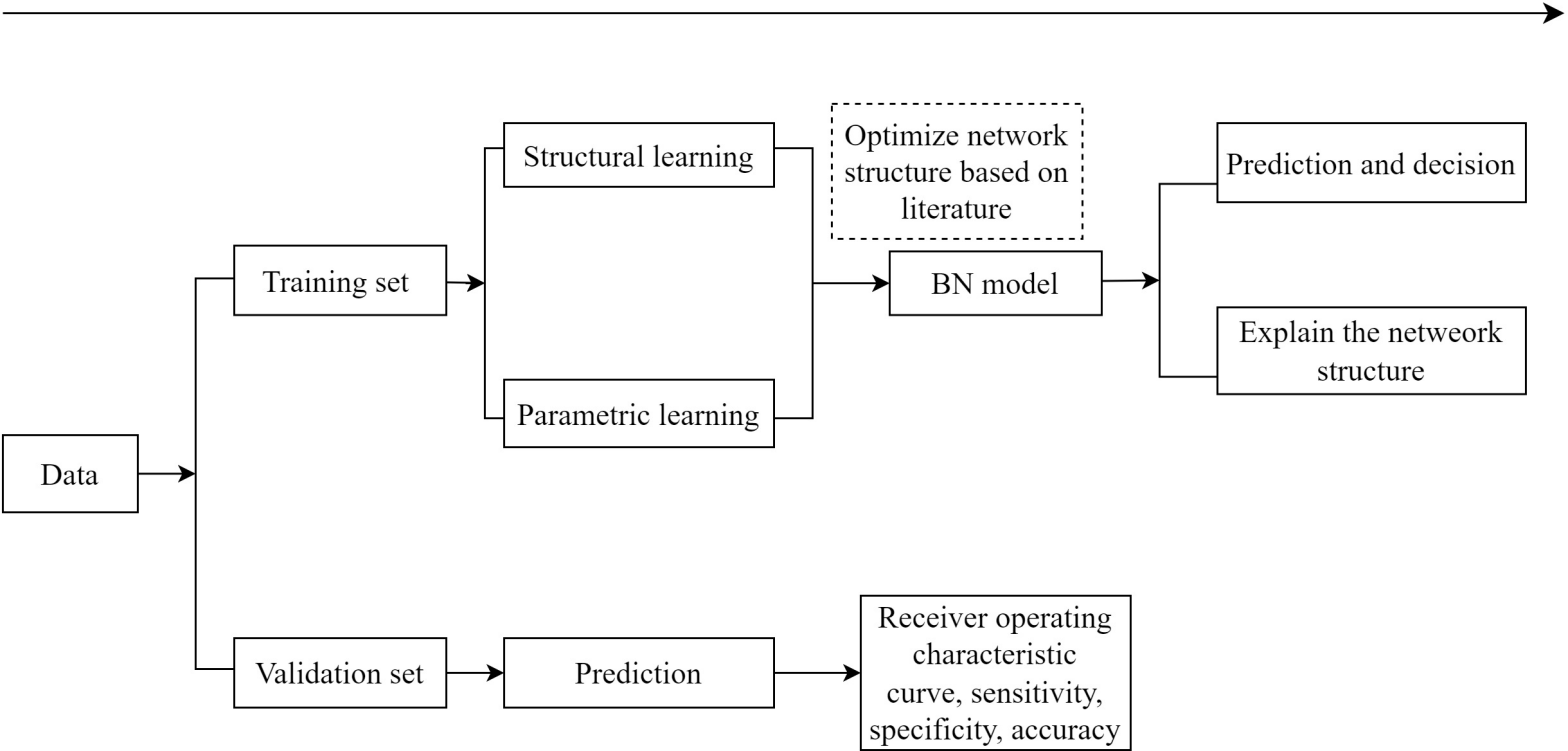
The flow diagram of Bayesian network model.

In the present study, diabetes was set as the deterministic node, and all of the 25 variables (**Supplementary Table 1**) included in constructing the initial network were selected on the basis of the literature (3, 16, 17) and data availability in the present study. The flow diagram of BN learning is shown in **Figure 1**. The first step was to obtain an initial network structure using the structural expectation maximization algorithm by setting blacklist of arcs (those included variables cannot point to age or sex, and variables occurring later in time sequence cannot point to baseline variables that occurred earlier, such as new-onset diabetes and baseline IFG) and whitelist of arcs [those included conditional dependencies between variables consistent with common sense or the prior knowledge, such as family history →IFG, drinking → high-density lipoprotein cholesterol (HDL-C), WC →hypertension, and smoking →body mass index (BMI)] (18–21). We performed bootstrap resampling on the dataset to obtain multiple training sets (200 resampling in this study), obtained 200 network structures, calculated the frequency of each directed arc in the 200 network structures, and kept the directed arcs with frequencies higher than 60% to obtain the average network structure. The second step was to optimize network structure based on previous knowledge (i.e., by removing illogical arcs and adjusting illogical orientation of arcs). In the third step, the Bayesian parameter estimation was used for parameters learning, and conditional probability tables were obtained to quantify the probability of each event (a node) based on all possible combinations of its parent nodes’ values. The last step was to evaluate the performance of the BN model.

### Validation

To obtain a robust estimate from a training model, we used 80% of the data for training set and 20% for validation set. A post-test probability table of the deterministic node with predicted risk was generated on the basis of the information from its Markov blanket. We performed five-fold cross-validation and calculated the probability threshold of the deterministic node for each validation fold. The basic idea of the five-fold cross-validation method is to group the original data into a training set and a validation set by a five-time loop. We calculated the averages area under the receiver operating characteristic curve (AUC), sensitivity, specificity, and predictive accuracy based on the five validation datasets to assess the performance of the established BN. The probability of diabetes was predicted in the validation dataset based on parameters estimated from training dataset, and the probability threshold for the validation dataset was calculated by maximizing the Youden index in the receiver operating characteristic curve analysis. Participants in the validation dataset were further classified into the new-onset diabetes and non-diabetes groups according to whether the probability estimates of the node diabetes surpassed the threshold. Sensitivity implies the ability of a model in identifying a patient as a positive result, specificity implies the ability of a model in identifying a non-patient as a negative result, AUC is a comprehensive index that integrates a model’s sensitivity and specificity, and accuracy implies the ability of a model in correctly discriminating patients from non-patients.

Forward and backward stepwise logistic regression model was used to identify risk factors for diabetes based on the dataset imputed by BN model, and optimal models were selected on the basis of the minimum Akaike information criterion value. As forward and backward stepwise methods generated the same results, we presented results from the forward models. Forced entry of the predictors (parent nodes) of diabetes in the BN into a logistic regression was used to construct a diabetes prediction model, and its prediction performance was compared with that of the BN model. Furthermore, as the prevalence of smoking or frequent alcohol use is much lower in Chinese women than men, we also conducted sensitivity analysis for each sex separately. We constructed BN models using the “bnlearn” package in R. All statistical analyses were done using R (version 4.0.0, https://www.r-project.org/). The code has been added to the **Supplementary Material**.

## Results

Of the 18,105 participants who returned for followed-up examination, 2,123 with baseline diabetes and 48 with incomplete data were excluded, giving 15,934 participants in the present (72.9% women) study. **Table 1** shows that 1,302 (8.17%) developed diabetes during an average follow-up of 4.1 (interquartile range = 3.34–4.49) years. At baseline, the mean (standard deviation) age was 61.0 (6.9) years. Participants who were older, had primary or below education; were non-drinkers (never and former); had daytime napping and snoring; had hypertension, lipid-lowering drug use, and family history of diabetes; had higher heart rate, BMI, WC, waist-to-hip ratio (WHR), triglycerides (TG), low-density lipoprotein cholesterol (LDL-C), and IFG; and had lower HDL-C had greater proportion of new-onset diabetes (P from<0.001 to 0.04).

**Table 1:** Percentage of new-onset diabetes at follow-up during 2008–2012 by baseline demographic and clinical characteristics (25 variables) in all participants.

Variables, N (%)	New-onset diabetes			
	No (%)	Yes (%)	Total	P-value
**Number (%)**	14,632 (91.83)	1,302 (8.17)	15,934	
**Sex**				0.44
Women	10,649 (91.72)	961 (8.28)	11,610	
Men	3,983 (92.11)	341 (7.89)	4,324	
**Age, years**				<0.001
<55	3,465 (94.93)	185 (5.07)	3,650	
55–65	6,958 (91.69)	631 (8.31)	7,589	
≥65	4,209 (89.65)	486 (10.35)	4,695	
**Education**				<0.001
Primary school or below	5,496 (90.29)	591 (9.71)	6,087	
Middle school	7,824 (93.01)	588 (6.99)	8,412	
College or above	1,308 (91.40)	123 (8.60)	1,431	
**Occupation**				0.18
Manual	8,777 (91.69)	795 (8.31)	9,572	
Non-manual	3,509 (91.59)	322 (8.41)	3,831	
Others	2,261 (92.78)	176 (7.22)	2,437	
**Family income, CNY/year**				0.53
<10,000	724 (91.53)	67 (8.47)	791	
10,000–49,999	7,993 (92.07)	688 (7.93)	8,681	
≥50,000	2,700 (92.59)	216 (7.41)	2,916	
**Smoking**				0.24
Never smokers	11,986 (91.71)	1,083 (8.29)	13,069	
Former smokers	1,197 (91.65)	109 (8.35)	1,306	
Current smokers	1,422 (92.94)	108 (7.06)	1,530	
**Drinking**				0.02
Never drinkers	8,821 (91.24)	847 (8.76)	9,668	
Former drinkers	311 (91.20)	30 (8.80)	341	
Current drinkers	4,344 (92.60)	347 (7.40)	4,691	
**Physical activity**				0.054
Inactive	1,257 (93.53)	87 (6.47)	1,344	
Moderately active	5,722 (91.55)	528 (8.45)	6,250	
Active	7,653 (91.76)	687 (8.24)	8,340	
**Insomnia**				0.39
No	12,091 (91.77)	1,085 (8.23)	13,176	
Yes	2,466 (92.29)	206 (7.71)	2,672	
**Daytime napping**				0.007
No	5,246 (92.65)	416 (7.35)	5,662	
Yes	9,308 (91.41)	875 (8.59)	10,183	
**Snoring**				<0.001
No	5,538 (93.11)	410 (6.89)	5,948	
Yes	6,155 (90.66)	634 (9.34)	6,789	
Do not know	2,862 (92.06)	247 (7.94)	3,109	
**Current health status compared with others**				0.13
Good	3,800 (92.50)	308 (7.50)	4,108	
Average	9,362 (91.53)	866 (8.47)	10,228	
Poor	1,398 (92.28)	117 (7.72)	1,515	
**Self-reported general health**				
Better	209 (90.09)	23 (9.91)	232	0.32
About the same	11,812 (91.84)	1,050 (8.16)	12,862	
Poor	2,201 (91.75)	198 (8.25)	2,399	
Worse	29 (100)	0 (0)	29	
**Hypertension**				<0.001
No	9,199 (94.61)	524 (5.39)	9,723	
Yes	5,357 (87.45)	769 (12.55)	6,126	
**Heart rate, beats/min**				<0.001
<60	748 (93.27)	54 (6.73)	802	
60–99	13,536 (91.91)	1,192 (8.09)	14,728	
≥100	348 (86.14)	56 (13.86)	404	
**Lipid-lowering drugs**				<0.001
No	13,126 (92.06)	1,132 (7.94)	14,258	
Yes	473 (85.69)	79 (14.31)	552	
**Self-reported coronary heart disease**				0.24
No	14,142 (91.90)	1,246 (8.10)	15,388	
Yes	418 (90.28)	45 (9.72)	463	
**Family history of diabetes**				<0.001
No	13,047 (92.26)	1,095 (7.74)	14,142	
Yes	1585 (88.45)	207 (11.55)	1,792	
**BMI, kg/m^2^**				<0.001
<25.0	10,251 (94.03)	651 (5.97)	10,902	
25.0–29.9	3,915 (87.64)	552 (12.36)	4,467	
≥30.0	437 (81.84)	97 (18.16)	534	
**Waist circumference, cm**				<0.001
<90 in men/<80 in women	10,283 (94.12)	642 (5.88)	10,925	
≥90 in men/≥80 in women	4,307 (86.80)	655 (13.20)	4,962	
**Waist-to-hip ratio**				<0.001
<0.9	11,017 (93.59)	755 (6.41)	11,772	
≥0.9	3,564 (86.82)	541 (13.18)	4,105	
**Triglycerides, mmol/L**				<0.001
<1.7	10,166 (93.93)	657 (6.07)	10,823	
≥1.7	4,441 (87.34)	644 (12.66)	5,085	
**High-density lipoprotein-cholesterol, mmol/L**				<0.001
<1.0	326 (85.12)	57 (14.88)	383	
≥1.0	14,279 (91.99)	1,244 (8.01)	15,523	
**Low-density lipoprotein cholesterol, mmol/L**				0.04
<3.4	8,866 (92.18)	752 (7.82)	9,618	
≥3.4	5,707 (91.27)	546 (8.73)	6,253	
**Impaired fasting glucose**				<0.001
No	10,872 (95.67)	492 (4.33)	11,364	
Yes	3,675 (82.64)	772 (17.36)	4,447	

### Construction of BN model and assignments

The deterministic node was defined as new-onset diabetes (yes/no). During construction of the initial BN, a total of 24 categorical variables were included. We applied expectation maximization algorithm to construct the structure and further optimized it based on current knowledge, by removing six illogical arcs ([age → family_history], [WC → education], [education → family_history], [IFG → physical_activity], [diabetes → TG], [drinking → LDL_C] (18), adjusting the illogical orientation of one arc ([TG → rxlipid (lipid-lowering drugs)]). Detailed strength of the conditional dependence relationships between nodes can be found in **Supplementary Table 2**.

**Figure 2:**
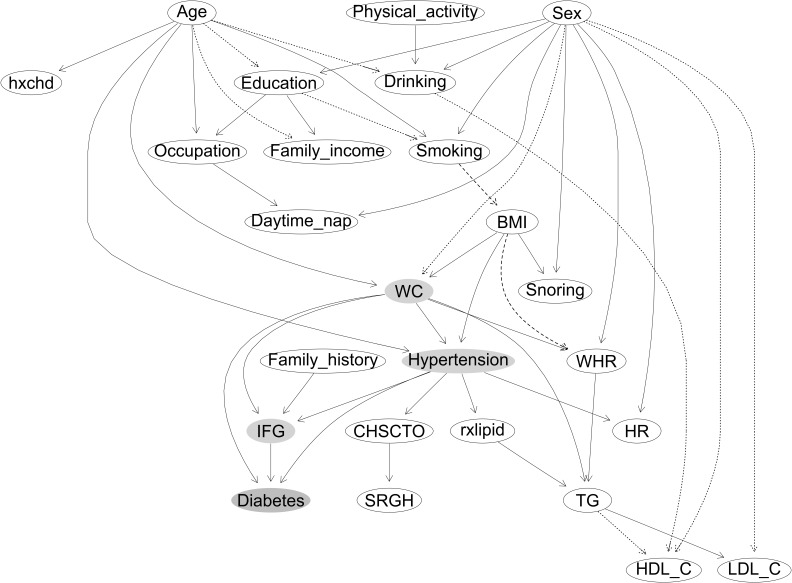
The constructed Bayesian network model of new-onset diabetes. (1) Labeled ovals represent nodes; arrows (arcs) represent (likely) causal relationships. Node in orange represents the deterministic node, and nodes in blue represent the nodes in the Markov blanket of the deterministic node. Arcs between the nodes with solid lines indicate positive association, and dotted line indicates negative associations. Arcs between the nodes with dashed lines indicate that, compared with the never smokers, former smokers had a higher probability of having greater BMI, whereas current smokers had a lower probability. Compared with those with BMI low than 25 kg/m^2^, participants with BMI above 25 and less than 30 kg/m^2^ had a higher probability of having greater WHR, whereas those with BMI above 30 kg/m^2^ had a lower probability. (2) Variables considered and/or tested were based on previous studies in the literature and data available in the present study, as follows: sex; age; education; occupation; family income; smoking; drinking; physical activity; daytime nap, daytime napping; snoring; CHSCTO, current health status compared with others; SRGN, self-reported general health; hypertension; HR, heat rate; rxlipid, lipid lowering drugs; hxchd, self-reported coronary heart disease; family history, family history of diabetes; BMI, body mass index; WC, waist circumference; WHR, waist-to-hip ratio; TG, triglycerides; HDL-C, high-density lipoprotein-cholesterol; LDL-C, low-density lipoprotein cholesterol; IFG, impaired fasting glucose.

**Figure 2** shows that most of the parent nodes were positively associated with their child nodes, except the following associations that were negative: sex → WC, sex → HDL-C, sex → LDL-C, age → drinking, age → family income, education → smoking, drinking→ HDL-C, and TG → HDL-C. Compared with the never smokers, the former smokers had a higher probability of having greater BMI, whereas the current smokers had a lower probability (smoking → BMI). Compared with those with BMI low than 25 kg/m^2^, participants with BMI above 25 and less than 30 kg/m^2^ had a higher probability of having greater WHR, whereas those with BMI above 30 kg/m^2^ had a lower probability (BMI → WHR). The three parent nodes of the new-onset diabetes indicating likely casual associations were hypertension, IFG, and WC. Sex was indirectly associated with BMI (sex → smoking→ BMI), and BMI was indirectly associated with diabetes through WC (BMI → WC) and through hypertension (BMI → hypertension). Hence, smoking was indirectly associated with diabetes through BMI and then through WC and hypertension. Age was directly associated with hypertension and WC and indirectly associated with BMI (age → education → smoking→ BMI), which led to a higher risk of diabetes. The network structure also showed many pathways between parent nodes and their child nodes, such as the associations among three types of cholesterol levels (TG → LDL-C, and TG → HDL-C/negative).

**Supplementary Figure 1** shows BN models by sex. In women, hypertension, IFG, and WC were likely casually associated with new-onset diabetes. Age was associated with hypertension, smoking, and WC. Smoking was associated with BMI, and BMI was indirectly associated with diabetes through WC and hypertension (BMI →WC, and BMI →hypertension) (same as **Figure 2**).

In men, hypertension and IFG were likely casually associated with diabetes. WC was indirectly associated with new-onset diabetes WHR and then IFG (WC→WHR→ IFG) and through hypertension (WC→ hypertension). Age was directly associated with hypertension and indirectly associated with smoking (age → education/negative → smoking/negative). Smoking was associated with BMI, and BMI was indirectly associated with diabetes through WC, WHR, and then IFG (BMI → WC → WHR → IFG) and through hypertension (BMI → hypertension).

### Posttest probability table of the deterministic node

**Table 2** shows that the post-test probability of diabetes in participants with hypertension only was 0.059. In those with hypertension and IFG, the probability was 0.157 and increased to 0.275 [95% confidence interval (CI): 0.272–0.276] if participants also had a higher WC (≥ 90 cm in men or ≥ 80 cm in women) (**Supplementary Figure 2**). In stratified analysis by sex, the post-test probability table of the deterministic nodes showed the same three predictors in women, but only hypertension and IFG (but not WC) were included in men (**Supplementary Table 3**; **Supplementary Figure 3**).

**Table 2:** Post-test probability table of the deterministic nodes in all participants.

Hypertension	IFG	WC, cm	Post-test probability diabetes	95% CI
No	No	<90 in men/<80 in women	0.021	(0.020–0.021)
No	No	≥90 in men/≥80 in women	0.043	(0.442–0.044)
Yes	No	<90 in men/<80 in women	0.059	(0.058–0.060)
Yes	No	≥90 in men/≥80 in women	0.102	(0.099–0.102)
No	Yes	<90 in men/<80 in women	0.121	(0.118–0.121)
No	Yes	≥90 in men/≥80 in women	0.168	(0.166–0.171)
Yes	Yes	<90 in men/<80 in women	0.157	(0.155–0159)
Yes	Yes	≥90 in men/≥80 in women	0.275	(0.272–0.276)

WC, waist circumference; IFG, impaired fasting glucose; CI, confidence interval.

### Performance of the BN risk prediction

The AUC of BN risk prediction model, first calculated in the training set and then verified in the validation set, showed satisfactory discrimination capability. The average AUC was 0.748 (95% CI: 0.742–0.755) in the training set. The average AUC was 0.746 (95% CI: 0.732–0.760), sensitivity was 0.727 (95% CI: 0.703–0.752), specificity was 0.660 (95% CI: 0.652–0.667), and accuracy was 0.665 (95% CI: 0.658–0.672) in the validation set. The highest performance of the BN model in the five-fold cross-validation reached 0.758 for AUC, 0.628 for sensitivity, 0.773 for specificity, and 0.762 for accuracy. In women, the AUC of BN model was 0.745 (95% CI: 0.729–0.761), sensitivity was 0.730 (95% CI: 0.702–0.759), specificity was 0.655 (95% CI: 0.646–0.664), and accuracy was 0.661 (95% CI: 0.653–0.670) in the validation set. In men, the AUC of BN model was 0.724 (95%CI: 0.697–0.752), sensitivity was 0.654 (95% CI: 0.603–0.704), specificity was 0.730 (95% CI: 0.716–0.744), and accuracy was 0.724 (95% CI: 0.710–0.737) in the validation set.

Using forward stepwise logistic regression model to identify predictors of new-onset diabetes based on the same set of 24 variables yielded 10 significant predictors (P< 0.05), with an AUC of 0.752 (95% CI: 0.723–0.782), sensitivity of 0.850, specificity of 0.548, and accuracy of 0.573 for the prediction model in the validation set (**Supplementary Table 4**). In women, the AUC of the prediction model including 11 factors from logistic regression was 0.803 (95% CI: 0.774–0.832), sensitivity was 0.833, specificity was 0.642, and accuracy was 0.658 (**Supplementary Table 5**). In men, the AUC of the prediction model including nine factors from logistic regression was 0.741 (95% CI: 0.686–0.796), sensitivity was 0.697, specificity was 0.734, and accuracy was 0.731 (**Supplementary Table 6**).

In addition, the AUC of the prediction model from forced entry logistic regression including the same three predictors of the BN model was 0.734 (95% CI: 0.703–0.764), the sensitivity was 0.604, the specificity was 0.745, and the accuracy was 0.734 (**Supplementary Table 7**). The high adjusted odds ratios (ORs) for IFG in women (3.90), men (4.51) and all (4.05) indicated that IFG was a strong predictor of new-onset diabetes.

## Discussion

We have reported the first diabetes risk prediction model by using BN model to integrate collinearity and complex interactions among multiple exposures and graphically revealing the associations (direct, indirect, and no) among 25 factors and new-onset diabetes. Three common risk factors (hypertension, IFG, and WC) were identified likely casual associations with new-onset diabetes, with a very high posttest probability of 27.5% (95% CI: 27.2%–27.6%) within about 4 years in participants with all three factors. Among the 64 existing conventional prediction models, the number of predictors in our BN model was among the smallest but with satisfactory performance (three predictors, AUC = 0.746). Moreover, as the BN model shows all the variables in one graph, it can provide useful insights for casual and non-causal pathways in disease etiology and mechanisms beyond those from conventional regression models and stepwise selection.

As the development of new-onset diabetes involves multiple factors which may play independent (direct or indirect) and/or synergistic roles, the directed arc of BN can also account for collinearity and interactions among multiple factors and provide a full network for the included factors. Our BN model identified three key factors of new-onset diabetes, of which higher systolic blood pressure and WC have also been identified as likely causes of diabetes in previous MR studies (22), and the other factor of IFG can be considered as pre-diabetes. Notably, we found that WC in all participants (and in women), rather than BMI, was directly associated with new-onset diabetes, which is consistent with a meta-analysis showing that WC was superior to BMI in predicting diabetes in women aged over 60 years (23). Moreover, the direct association of WC on IFG also consistently supports the detrimental effects of higher WC on hyperglycemia (24). Our stratified analysis showed that WC was likely casually associated with new-onset diabetes in women but not in men. In men, WC can be considered as an antecedent causal factor with indirect associations with diabetes through hypertension and through WHR and IFG. Although there is some evidence for the ethnic differences in obesity and disease risk (25), whether men or women are more susceptible to central obesity (i.e., higher WC) and its subsequent diabetic risk is yet to be confirmed. What and how different types and indicators of adiposity or obesity increase the risk of diabetes deserves further research on men and women separately.

Our results also showed other interesting associations or pathways. Family history of diabetes, WC, and hypertension were associated with IFG. Previous studies reported a two- to six-fold increased risk of diabetes associated with a family history of diabetes compared with those without (26–28). We found that family history of diabetes and lifestyle factors were independent of each other, suggesting that such family history was unlikely to play a role in adopting a healthy lifestyle in our participants who were older people. Moreover, our direct associations of hypertension with IFG and diabetes consistently support that higher blood pressure may play a causal role in the development of diabetes. Such adverse effects may be due to microvasculature dysfunction, leading to small vessel stenosis and progression of diabetic complications (29). As diabetes and hypertension share some common underlying pathology and risk factors, as well as harms (30), patients with hypertension but not diagnosed or having diabetes need more frequent periodic screening for diabetes or prediabetes (31).

An MR study showed likely causal associations of alcohol drinking with diabetes (32). However, our results do not support a causal effect of alcohol drinking on diabetes. Notably, inconsistent results were also reported in previous studies, with a U-shaped association reported in a meta-analysis of 38 observations studies (cohort, case-cohort, case-control, and nested case-control designs) (33) but a linear and positive association in an MR study (32). The discrepancy might be due to different ethnicities and exposures. For example, a previous study showed that the reductions in diabetic risk associated with moderate alcohol use were specific to women and non-Asian populations (33). Whether the alcohol–diabetes association varies by ethnicity and sex and, if so, the underlying mechanisms need to be explored.

The present BN investigation has shown that smoking (never, former, and current) was indirectly associated with diabetes in the total sample. In women and men, smoking was indirectly associated with diabetes, through the several major risk factors including WC or BMI and hypertension. A previous MR study showed a likely causal association of smoking (smoking initiation) with diabetes (34), and the US Surgeon General’s Reports concluded that smoking can cause diabetes (35). Our results showed that, compared with never smokers, former smokers had higher BMI, but current smokers had lower BMI than never smokers in all participants (and in men), suggesting that quitting smoking could lead to increased adiposity (36). This potential weight-increasing effect needs to be considered in future studies on the relationship between smoking and diabetes. However, causation based only on observational data is not definitive, and we have found no evidence from clinical trials that quitting smoking can reduce the risk of diabetes.

The predictive performance of our BN model was satisfactory, achieving 0.746 (95% CI: 0.732–0.760), 0.727, and 0.660 in AUC, sensitivity, and specificity, respectively. The AUC of BN model was slightly lower than (but with overlapping 95% CI) that from the conventional prediction model including 10 significant predictors from logistic regression (AUC = 0.752, 95% CI = 0.723–0.782), but the AUC estimate of the former was more precise (narrower 95% CI) than that of the latter. Moreover, the AUC from the conventional prediction model including the same set of three predictors was smaller (AUC = 0.734, 95% CI = 0.703–0.764) with overlapping but wider 95% CI. The results of the stratified analysis showed that the AUCs of BN and logistic model were 0.745 and 0.803 in women and were 0.724 and 0.741 in men, respectively. The BN models and the logistic regression models showed about the same AUC on average, but the logistic regression had higher AUC in the stratified models, suggesting that logistic regression might have suffered from confounding (such as Simpson’s paradox).

A 2011 meta-analysis of 43 prospective studies with predictive models showed three to 14 predictors with AUCs from 0.68 to 0.90.6. The smallest number of risk factors included in the prediction model was three, which was shown in only two of the included studies. One showed an AUC of 0.62 to 0.64 based on 3,094 Mauritian Indians including BMI, WC, and family history of diabetes (37). The other based on 3,817 French including predictors of WC and hypertension in both sexes, smoking in men, and family diabetes history in women showed AUC of 0.713 for men and 0.827 for women (38). Although we showed a lower AUC in women than this French study (38), our study of a larger sample and more comprehensive assessment of predictors should provide a more reliable and robust prediction. Moreover, we searched PubMed using keywords of “type 2 diabetes” AND predict* AND (model OR score OR equation) AND (prospective OR follow-up OR “follow up”) for diabetes prediction models published after the 2011 meta-analysis (6) from 01 March 2011 to 21 June 2021 and found 21 diabetes risk prediction models from 14 papers. These latter models showed AUCs from 0.66 to 0.90 with five to 17 predictors. Most of these studies included measurements of fasting glucose, BMI, and blood pressure in the prediction models, supporting the key roles of these factors in developing diabetes. Our study has provided a novel, simple but comparable (in terms of prediction performance) model for identifying individuals at risk of developing diabetes in the next 3–5 years and thus adds to the literature on diabetes prediction. As the predictors in our risk model can be easily measured and readily accessible and the established post-test probability table shows the probability of different combinations of the three factors explicitly, high-risk sub-groups can be identified for public health and clinical interventions.

BN shows likely causal pathways between factors and new-onset diabetes prospectively and among other factors (associations among baseline factors were cross-sectional) and unlikely causal relationships. Moreover, all previous prediction models using traditional regression did not provide information on causal pathways among predictors or risk factors, but conventional methods are simple and can show relative risks or ORs. Hence, results from both methods should be considered to facilitate a more thorough understanding of disease mechanisms and guide further research.

Our study had several limitations. First, we obtained an initial network structure using the structural expectation maximization algorithm. Further studies to clarify the difference of the initial network structure constructed by different learning algorithms are warranted. Second, we did not measure 2-h post-load glucose at baseline, and thus, some cases of diabetes at baseline might have been included in the analyses. However, as 2-h oral glucose tolerance test is much time-consuming and may not improve the discriminatory capacity substantially (39). Third, some known predictors of diabetes, such as HbA1c, insulin resistance, and the homeostatic model assessment β-cell index (40), were not included, but these risk factors are not routinely measured in primary care. Fourth, about 38% of the participants did not return for repeated examinations during follow-up. In our previous study, we found that older men with lower socioeconomic position, unhealthy lifestyles, and poorer health status tended not to return for follow-up examination. Thus, the diabetes incidence might have been underestimated if the non-participants had a higher risk of diabetes. Nevertheless, such follow-up rate was common in large cohorts with repeated measurements (41). Finally, as all participants were southern Chinese aged 50 years or more, survivor bias might limit the generalizability of the results.

## Conclusions

We have first reported a BN model in predicting new-onset diabetes with the smallest number of factors among existing models in the literature. BN yielded a more comprehensive figure showing graphically the inter-relations (direct, indirect, and no association) for multiple factors with diabetes than existing regression models. Further studies on the effectiveness of using simple diabetes prediction model to improve health outcomes are warranted.

## Data availability statement

The original contributions presented in the study are included in the article/**Supplementary Material**. Further inquiries can be directed to the corresponding authors.

## Ethics statement

Guangzhou Biobank Cohort Study was reviewed and approved by the Guangzhou Medical Ethics Committee of the Chinese Medical Association. The patients/participants provided their written informed consent to participate in this study.

## Author contributions

YW helped conceive the design of the study, analyzed the data, and draft the manuscript; LX helped conceive the design of the study, analyzed the data, and revised the article for critically important intellectual content. THL helped conceive the design of the study and revised the article for critically important intellectual content; WZ, CJ and YJ performed data collection; WZ and YH helped draft the manuscript; KKC helped conceive the design of the study and revised the article. All authors have read and approved the final version of the manuscript, and agree with the order of the presentation of the authors.

## Funding

This work was supported by the National Natural Science Foundation (Nos. 81941019, 81973150, and 81773543); the Natural Science Foundation of Guangdong (No. 2018A030313140); the Guangzhou Science and Technology Bureau, Guangzhou, China (No. 201704030132); the Major Infectious Disease Prevention and Control of the National Science and Technique Major Project (No. 2018ZX10715004); and the University of Birmingham, UK.

## Conflict of interest

The authors declare that the research was conducted in the absence of any commercial or financial relationships that could be construed as a potential conflict of interest.

## Publisher’s note

All claims expressed in this article are solely those of the authors and do not necessarily represent those of their affiliated organizations, or those of the publisher, the editors and the reviewers. Any product that may be evaluated in this article, or claim that may be made by its manufacturer, is not guaranteed or endorsed by the publisher.

## References

[1] World Health Organization. E. coli (2022). Available at: https://www.who.int/news-room/fact-sheets/detail/diabetes (Accessed March 25, 2022).

[2] IDF diabetes atlas, in: 2019. e. coli (2022). Available at: https://diabetesatlas.org/en (Accessed March 25, 2022).

[3] Bellou V Belbasis L Tzoulaki I Evangelou E. Risk factors for type 2 diabetes mellitus: an exposure-wide umbrella review of meta-analyses. PloS One (2018) 13(3):e194127. doi: 10.1371/journal.pone.0194127 PMC5860745 29558518

[4] Chen L Magliano DJ Zimmet PZ. The worldwide epidemiology of type 2 diabetes mellitus–present and future perspectives. Nat Rev Endocrinol (2011) 8(4):228–36. doi: 10.1038/nrendo.2011.183 22064493

[5] Xu L Jiang CQ Schooling CM Zhang WS Cheng KK Lam TH. Prediction of 4-year incident diabetes in older Chinese: recalibration of the framingham diabetes score on guangzhou biobank cohort study. Prev Med (2014) 69:63–8. doi: 10.1016/j.ypmed.2014.09.004 25239055

[6] Noble D Mathur R Dent T Meads C Greenhalgh T. Risk models and scores for type 2 diabetes: systematic review. BMJ (2011) 343:d7163. doi: 10.1136/bmj.d7163 22123912 PMC3225074

[7] Emdin CA Khera AV Kathiresan S. Mendelian randomization. JAMA (2017) 318(19):1925–6. doi: 10.1001/jama.2017.17219 29164242

[8] VanderWeele TJ Robins JM. Signed directed acyclic graphs for causal inference. Ser B Stat Methodol (2010) 72(1):111–27. doi: 10.1111/j.1467-9868.2009.00728.x PMC4239133 25419168

[9] Lucas PJ van der Gaag LC Abu-Hanna A. Bayesian Networks in biomedicine and health-care. Artif Intell Med (2004) 30(3):201–14. doi: 10.1016/j.artmed.2003.11.001 15081072

[10] Stockel D Schmidt F Trampert P Lenhof HP. CausalTrail: Testing hypothesis using causal Bayesian networks. F1000Res (2015) 4:ISCB Comm J–1520. doi: 10.12688/f1000research.7647.1 PMC4743151 26913195

[11] Tennant PWG Murray EJ Arnold KF Berrie L Fox MP Gadd SC. Use of directed acyclic graphs (DAGs) to identify confounders in applied health research: review and recommendations. Int J Epidemiol (2021) 50(2):620–32. doi: 10.1093/ije/dyaa213 PMC8128477 33330936

[12] Jiang C Thomas GN Lam TH Schooling CM Zhang W Lao X. Cohort profile: The guangzhou biobank cohort study, a guangzhou-Hong Kong-Birmingham collaboration. Int J Epidemiol (2006) 35(4):844–52. doi: 10.1093/ije/dyl131 16844769

[13] Forouhi NG Balkau B Borch-Johnsen K Dekker J Glumer C Qiao Q. The threshold for diagnosing impaired fasting glucose: a position statement by the European diabetes epidemiology group. Diabetologia (2006) 49(5):822–7. doi: 10.1007/s00125-006-0189-4 16525842

[14] American Diabetes Association. Diagnosis and classification of diabetes mellitus. Diabetes Care (2013) 36 Suppl 1(Suppl 1):S67–74. doi: 10.2337/dc13-S067 PMC3537273 23264425

[15] Arora P Boyne D Slater JJ Gupta A Brenner DR Druzdzel MJ. Bayesian Networks for risk prediction using real-world data: a tool for precision medicine. Value Health (2019) 22(4):439–45. doi: 10.1016/j.jval.2019.01.006 30975395

[16] Friedman N. The Bayesian structural EM algorithm. Proceedings of the Fourteenth conference on Uncertainty in artificial intelligence. Madison, Morgan Kaufmann Publishers Inc (1988). p. P129–138.

[17] Yuan S Larsson SC. An atlas on risk factors for type 2 diabetes: a wide-angled mendelian randomisation study. Diabetologia (2020) 63(11):2359–71. doi: 10.1007/s00125-020-05253-x PMC7527357 32895727

[18] Rosoff DB Davey SG Mehta N Clarke TK Lohoff FW. Evaluating the relationship between alcohol consumption, tobacco use, and cardiovascular disease: a multivariable mendelian randomization study. PloS Med (2020) 17(12):e1003410. doi: 10.1371/journal.pmed.1003410 33275596 PMC7717538

[19] Fall T Hägg S Ploner A Mägi R Fischer K Draisma HH. Age- and sex-specific causal effects of adiposity on cardiovascular risk factors. Diabetes (2015) 64(5):1841–52. doi: 10.2337/db14-0988 PMC4407863 25712996

[20] Chen Q Li L Yi J Huang K Shen R Wu R. Waist circumference increases risk of coronary heart disease: Evidence from a mendelian randomization study. Mol Genet Genomic Med (2020) 8(4):e1186. doi: 10.1002/mgg3.1186 32090477 PMC7196469

[21] Åsvold BO Bjørngaard JH Carslake D Gabrielsen ME Skorpen F Smith GD. Causal associations of tobacco smoking with cardiovascular risk factors: a mendelian randomization analysis of the HUNT study in Norway. Int J Epidemiol (2014) 43(5):1458–70. doi: 10.1093/ije/dyu113 24867305

[22] Li K Feng T Wang L Chen Y Zheng P Pan P. Causal associations of waist circumference and waist-to-hip ratio with type II diabetes mellitus: new evidence from mendelian randomization. Mol Genet Genomics (2021) 296(3):605–13. doi: 10.1007/s00438-020-01752-z 33629185

[23] Seo DC Choe S Torabi MR. Is waist circumference >/=102/88cm better than body mass index >/=30 to predict hypertension and diabetes development regardless of gender, age group, and race/ethnicity? meta-analysis. Prev Med (2017) 97:100–8. doi: 10.1016/j.ypmed.2017.01.012 28137662

[24] Zhao M Lin H Yuan Y Wang F Xi Y Wen LM. Prevalence of pre-diabetes and its associated risk factors in rural areas of ningbo, China. Int J Environ Res Public Health (2016) 13(8):808. doi: 10.3390/ijerph13080808 PMC4997494 27517947

[25] Ujcic-Voortman JK Bos G Baan CA Verhoeff AP Seidell JC. Obesity and body fat distribution: ethnic differences and the role of socio-economic status. Obes Facts (2011) 4(1):53–60. doi: 10.1159/000324555 21372611 PMC6444595

[26] Bjornholt JV Erikssen G Liestol K Jervell J Thaulow E Erikssen J. Type 2 diabetes and maternal family history: an impact beyond slow glucose removal rate and fasting hyperglycemia in low-risk individuals? results from 22.5 years of follow-up of healthy nondiabetic men. Diabetes Care (2000) 23(9):1255–9. doi: 10.2337/diacare.23.9 10977015

[27] Mitchell BD Valdez R Hazuda HP Haffner SM Monterrosa A Stern MP. Differences in the prevalence of diabetes and impaired glucose tolerance according to maternal or paternal history of diabetes. Diabetes Care (1993) 16(9):1262–7. doi: 10.2337/diacare.16.9.1262 8404430

[28] Wagner R Thorand B Osterhoff MA Müller G Böhm A Meisinger C. Family history of diabetes is associated with higher risk for prediabetes: a multicentre analysis from the German center for diabetes research. Diabetologia (2013) 56(10):2176–80. doi: 10.1007/s00125-013-3002-1 23979484

[29] Grossman A Grossman E. Blood pressure control in type 2 diabetic patients. Cardiovasc Diabetol (2017) 16(1):3. doi: 10.1186/s12933-016-0485-3 28056987 PMC5217560

[30] Cloutier L Lamarre-Cliche M. Hypertension in adults with type 2 diabetes: a review of blood pressure measurement methods, targets and therapy. Can J Diabetes (2018) 42(2):188–95. doi: 10.1016/j.jcjd.2018.01.012 29602406

[31] Cryer MJ Horani T DiPette DJ. Diabetes and hypertension: a comparative review of current guidelines. J Clin Hypertens (Greenwich) (2016) 18(2):95–100. doi: 10.1111/jch.12638 26234374 PMC8031521

[32] Peng M Zhang J Zeng T Hu X Min J Tian S. Alcohol consumption and diabetes risk in a Chinese population: a mendelian randomization analysis. Addiction (2019) 114(3):436–49. doi: 10.1111/add.14475 30326548

[33] Knott C Bell S Britton A. Alcohol consumption and the risk of type 2 diabetes: a systematic review and dose-response meta-analysis of more than 1.9 million individuals from 38 observational studies. Diabetes Care (2015) 38(9):1804–12. doi: 10.2337/dc15-0710 26294775

[34] Yuan S Larsson SC. A causal relationship between cigarette smoking and type 2 diabetes mellitus: A mendelian randomization study. Sci Rep (2019) 9(1):19342. doi: 10.1038/s41598-019-56014-9 31852999 PMC6920406

[35] National Center for Chronic Disease Prevention and Health Promotion (US) Office on Smoking and Health. The health consequences of smoking–50 years of progress: A report of the surgeon general. Atlanta (GA: Centers for Disease Control and Prevention 2014) (US. Available at: https://www.ncbi.nlm.nih.gov/books/NBK179276/. 24455788

[36] Jain P Danaei G Robins JM Manson JE Hernán MA. Smoking cessation and long-term weight gain in the framingham heart study: an application of the parametric g-formula for a continuous outcome. Eur J Epidemiol (2016) 31(12):1223–9. doi: 10.1007/s10654-016-0200-4 PMC5759957 27704230

[37] Gao WG Qiao Q Pitkäniemi J Wild S Magliano D Shaw J. Risk prediction models for the development of diabetes in Mauritian indians. Diabetes Med (2009) 26(10):996–1002. doi: 10.1111/j.1464-5491.2009.02810.x 19900231

[38] Balkau B Lange C Fezeu L Tichet J de Lauzon-Guillain B Czernichow S. Predicting diabetes: clinical, biological, and genetic approaches: data from the epidemiological study on the insulin resistance syndrome (DESIR). Diabetes Care (2008) 31(10):2056–61. doi: 10.2337/dc08-0368 PMC2551654 18689695

[39] Gao WG Dong YH Pang ZC Nan HR Wang SJ Ren J. A simple Chinese risk score for undiagnosed diabetes. Diabetes Med (2010) 27(3):274–81. doi: 10.1111/j.1464-5491.2010.02943.x 20536489

[40] Gray LJ Taub NA Khunti K Gardiner E Hiles S Webb DR. The Leicester risk assessment score for detecting undiagnosed type 2 diabetes and impaired glucose regulation for use in a multiethnic UK setting. Diabetes Med (2010) 27(8):887–95. doi: 10.1111/j.1464-5491.2010.03037.x 20653746

[41] Kristman V Manno M Cote P. Loss to follow-up in cohort studies: how much is too much? Eur J Epidemiol (2004) 19(8):751–60. doi: 10.1023/b:ejep.0000036568.02655.f8 15469032

