# Supplementary material for: A Bayesian network model of new-onset diabetes in older Chinese: The Guangzhou biobank cohort study
Source: Front Endocrinol (Lausanne). 2022 Aug 3;13:916851. doi: 10.3389/fendo.2022.916851 (PMC9382298; doi:10.3389/fendo.2022.916851)
Supplement: Supplementary file 1 [file DataSheet_1.docx]

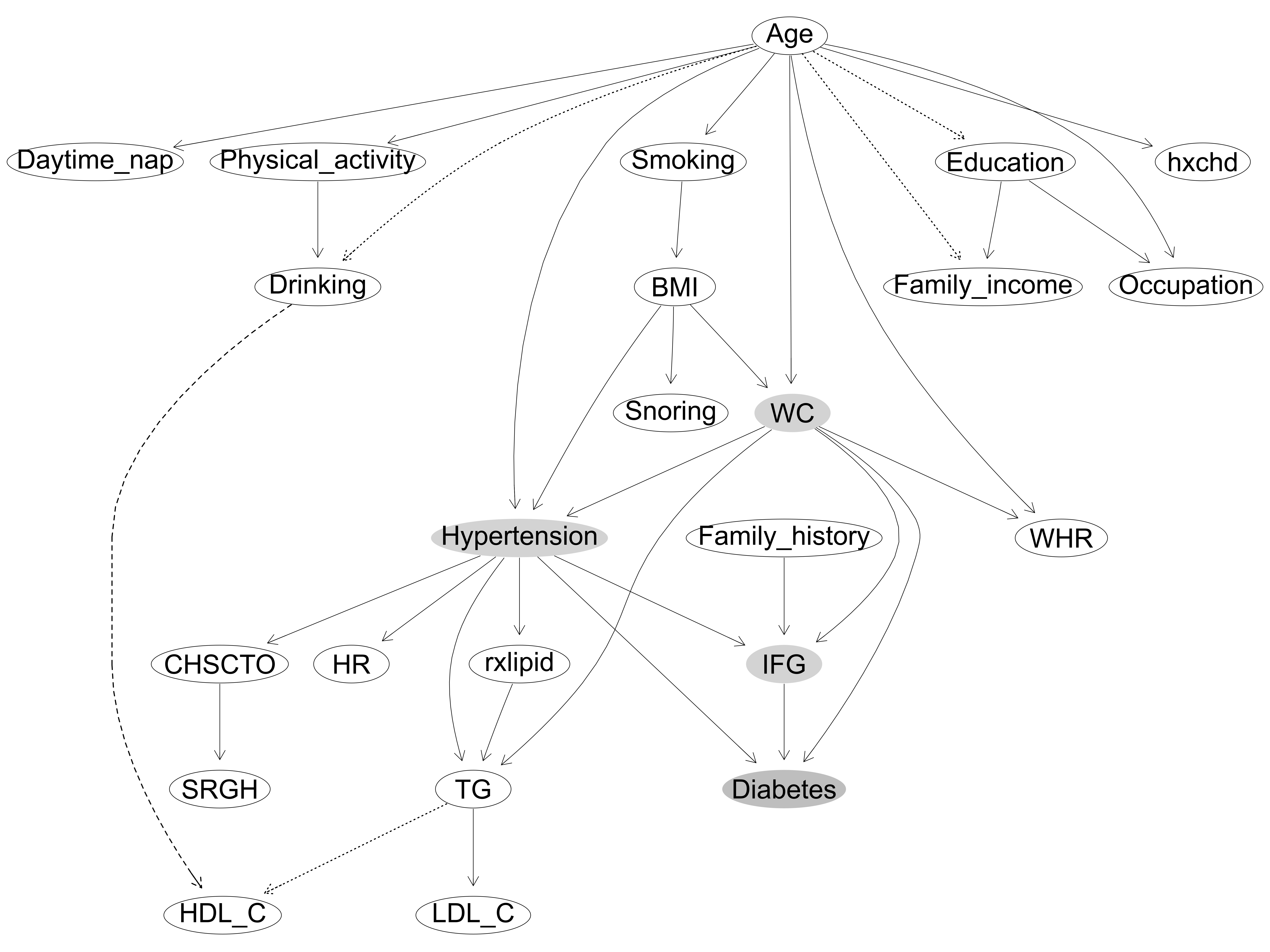


1. Women


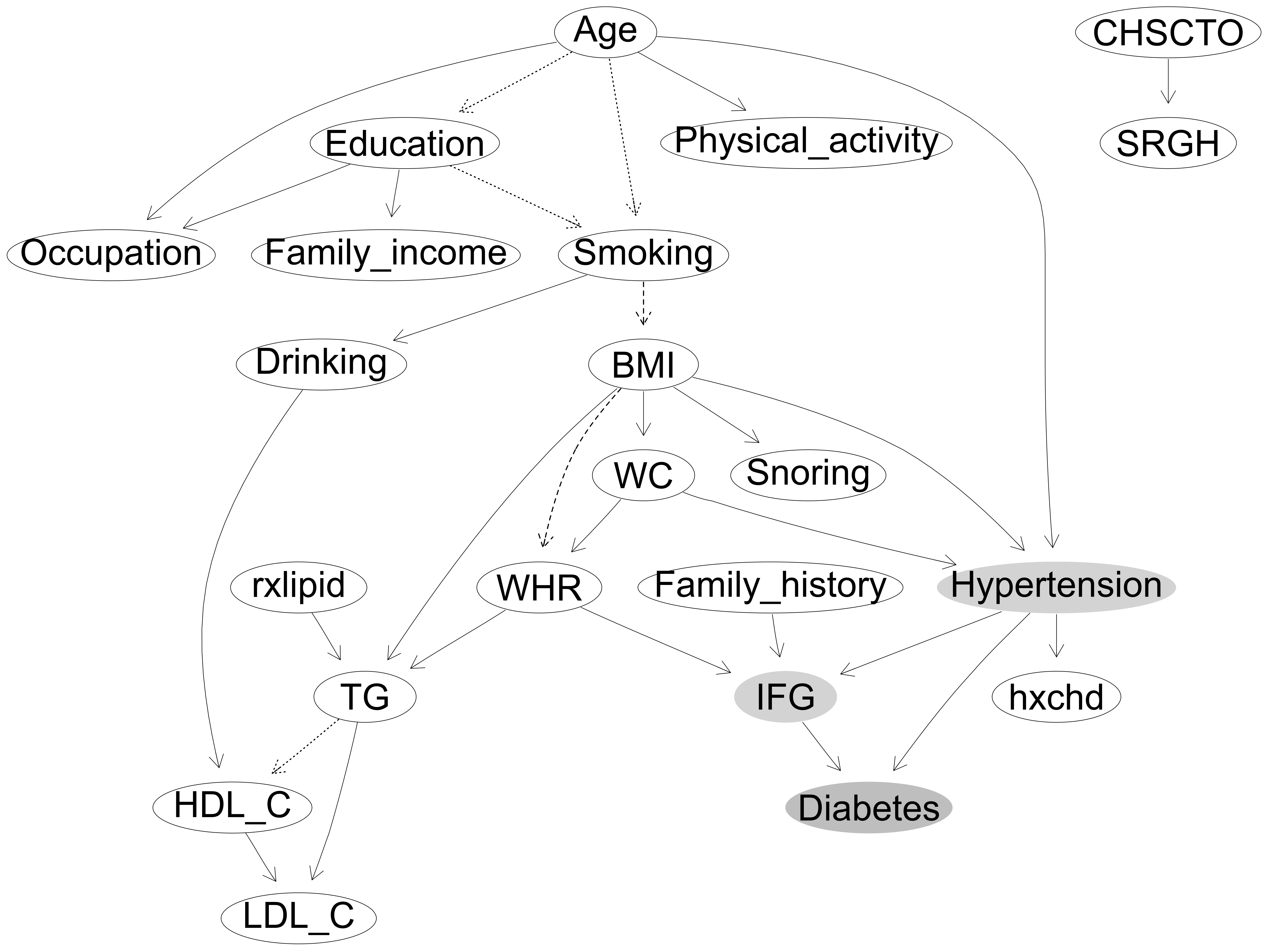


(b) Men

Supplementary figure 1: The constructed Bayesian network model of new-onset diabetes in women (a) and men (b).

Note:

1. Labelled ovals represent nodes; arrows (arcs) represent (likely) causal relationships. Node in orange represents the deterministic node and nodes in blue represent the nodes in the Markov blanket of the deterministic node. Arcs between the nodes with solid lines indicate positive association, and dotted lines indicate negative associations. Arcs between the nodes with dashed lines indicate that compared with the never smokers, former smokers had a higher probability of having greater BMI whilst current smokers had a lower probability in men. Compared with those with BMI low than 25 kg/m^2^, participants with BMI above 25 and less than 30 kg/m^2^ had a higher probability of having greater WHR, whilst those with BMI above 30 kg/m^2^ had a lower probability in men.
2. Variables considered and/or tested were based on previous studies in the literature and data available in the present study, as follows: Sex; Age; Education; Occupation; Family income; Smoking; Drinking; Physical activity; Insomnia; Daytime nap=daytime napping; Snoring; CHSCTO=current health status compared to others; SRGN=self-reported general health; Hypertension; HR=heat rate; rxlipid=lipid lowering drugs; hxchd=self-reported coronary heart disease; Family history=family history of diabetes; BMI=body mass index; WC=waist circumference; WHR=waist-to-hip ratio; TG=triglycerides; HDL-C=high-density lipoprotein-cholesterol; LDL-C=low-density lipoprotein cholesterol; IFG=impaired fasting glucose.


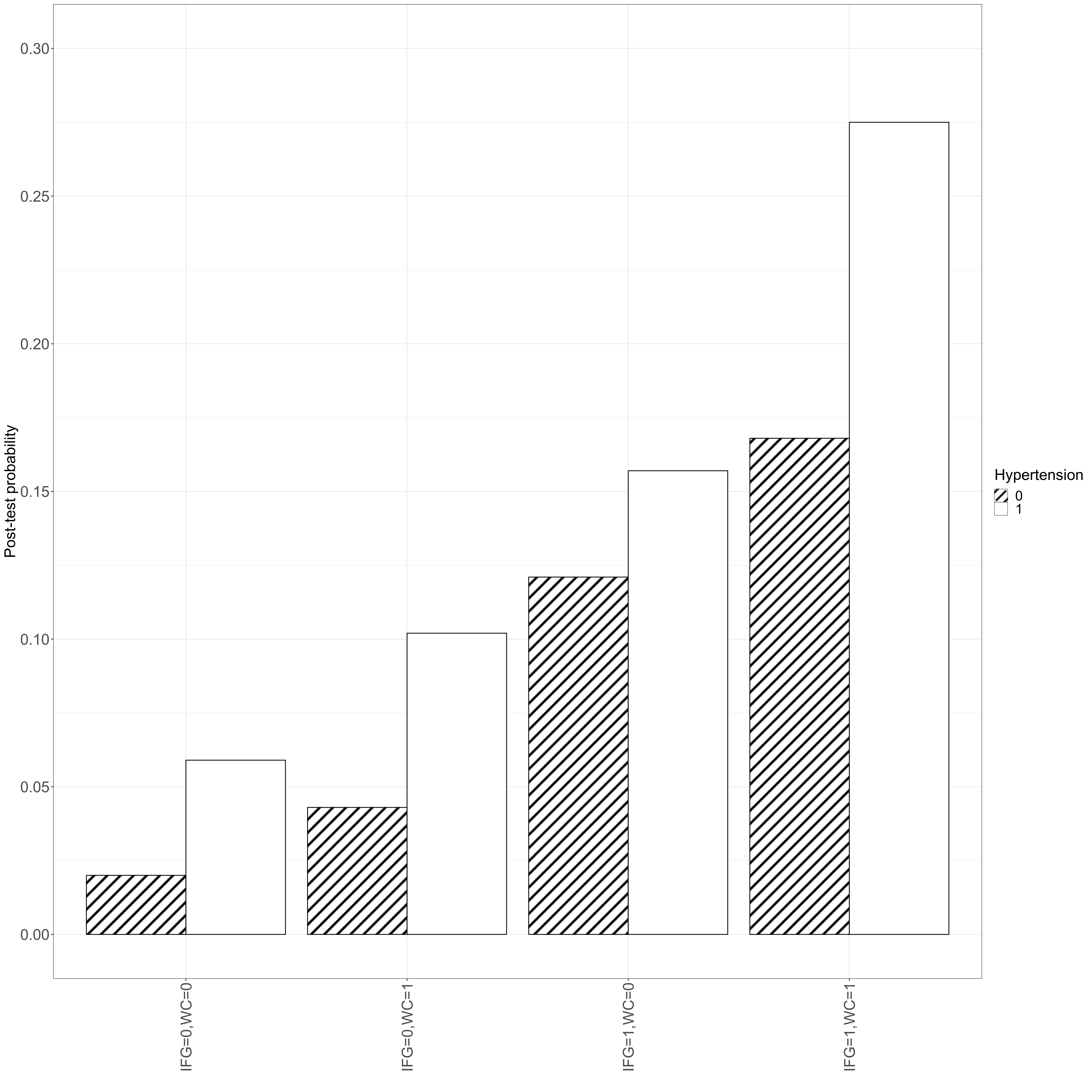


Supplementary figure 2. Post-test Probability of new-onset diabetes in all participants

Note: IFG, impaired fasting glucose; WC, waist circumference

Hypertension, No=0, Yes=1; WC, <90 cm in men or <80 cm in women =0, ≥90 cm in men or ≥80 cm in women=1; IFG, No=0, Yes=1.


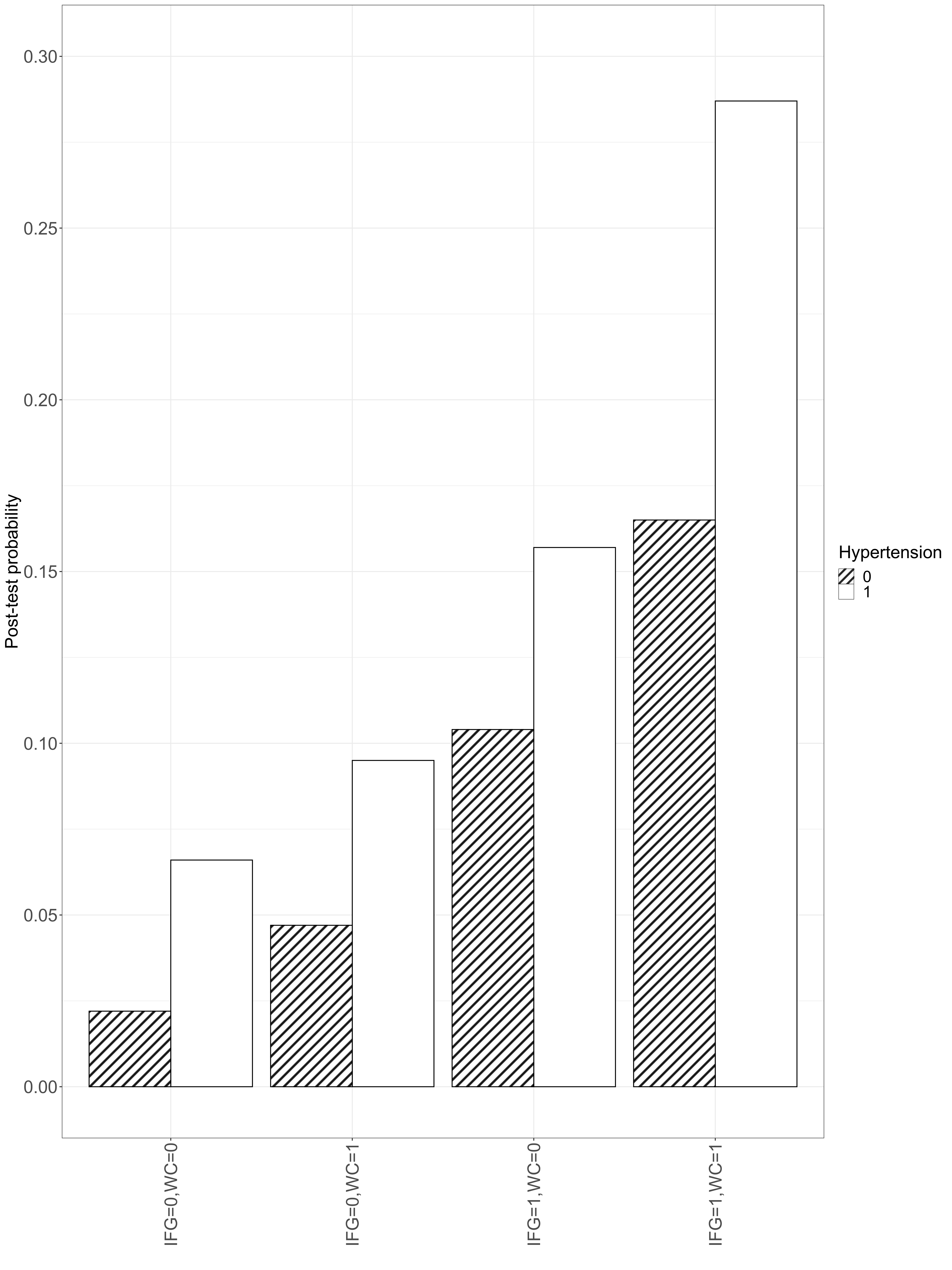


1. Women


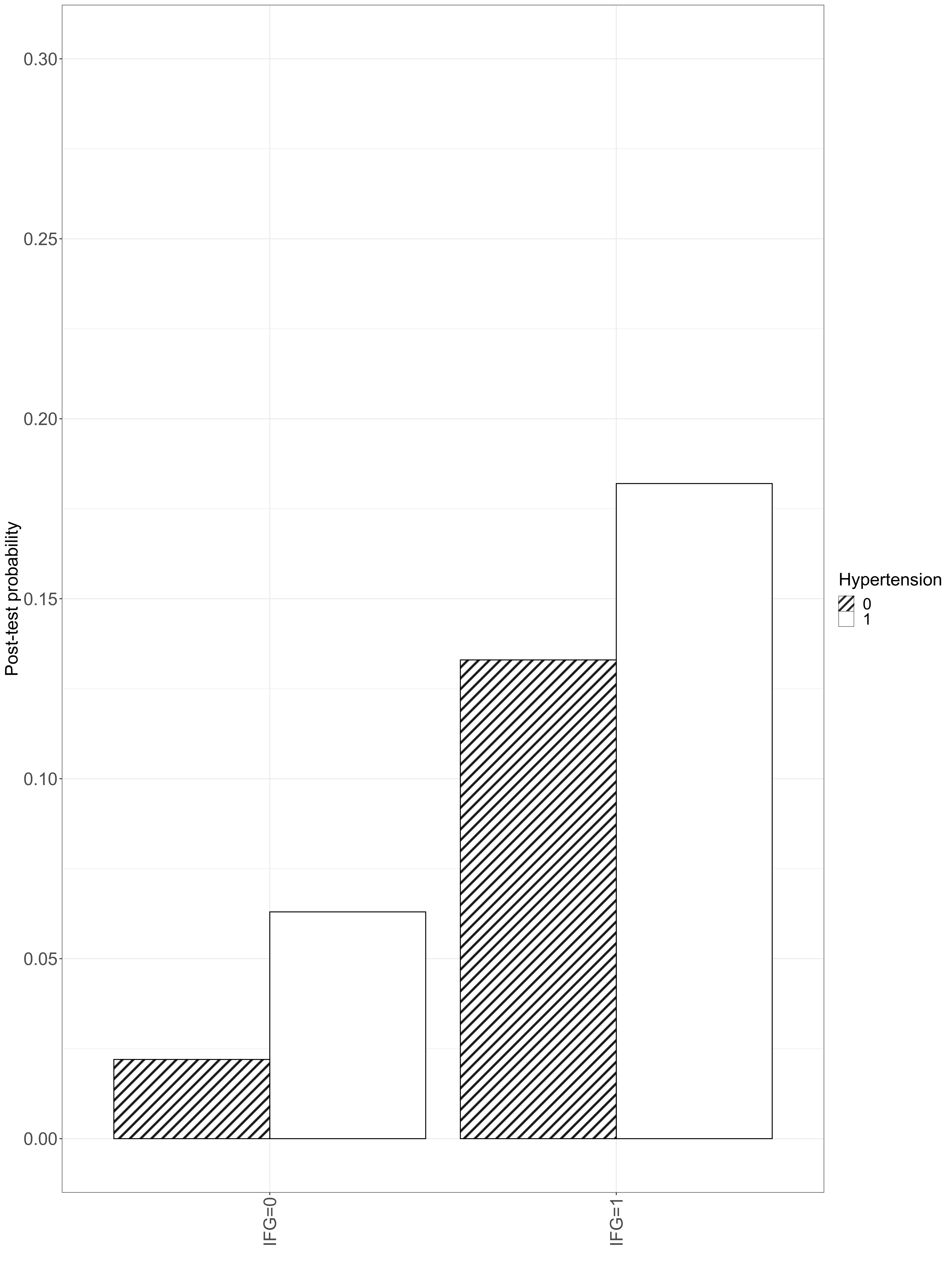


1. Men

Supplementary figure 3. Post-test Probability of new-onset diabetes by sex ((a) women, (b) men)

Note: IFG, impaired fasting glucose; WC, waist circumference

Hypertension, No=0, Yes=1; WC, <90 cm in men or <80 cm in women =0, ≥90 cm in men or ≥80 cm in women=1; IFG, No=0, Yes=1.

Supplementary table 1. Variables and assignment for BN model in all participants

| Variables | Code | Assignment |
| --- | --- | --- |
| Sex | Sex | Women=0, Men=1 |
| Age, years | Age | <55=0, 55-64=1, ≥65=2 |
| Education | Education | Primary=0, Middle school=1, College=2 |
| Occupation | Occupation | Manual=0, Non-manual=1, Others=2 |
| Family income | Family income | <10000 CNY/year =0, 10000-49999 CNY/year =1, ≥50000 CNY/year =2 |
| Smoking | Smoking | Never=0, Former smoker=1, Current smoker=2 |
| Drinking | Drinking | Never=0, Former drinker=1, Current drinker=2 |
| Physical activity | Physical activity | Inactive=0, Moderate=1, Active=2 |
| Insomnia | Insomnia | No=0, Yes=1 |
| Daytime napping | Daytime nap | No=0, Yes=1 |
| Snoring | Snoring | No=0, Yes=1, Don’t know =2 |
| Current health status compared to others | CHSCTO | Good=0, Average=1, Poor=2 |
| Self-reported general health | SRGH | Better=0, About the same=1, Poor=2, Worse=3 |
| Hypertension | Hypertension | No=0, Yes=1 |
| Heart rate | Heart rate | <60 beats/min=0, 60-99 beats/min =1, ≥100 beats/min =2 |
| Lipid lowering drugs | rxlipid | No=0, Yes=1 |
| Self-reported coronary heart disease | hxchd | No=0, Yes=1 |
| Family history of diabetes | Family history | No=0, Yes=1 |
| Body mass index | BMI | <25.0 kg/m^2^=0, 25.0-29.9 kg/m^2^=1, ≥30.0 kg/m^2^=2 |
| Waist circumference | WC | <90 cm in men or <80 cm in women =0, ≥90/80 cm in men or ≥80 cm in women=1 |
| Waist-to-hip ratio | WHR | <0.9=0, ≥0.9=1 |
| Triglycerides | TG | <1.7 mmol/L =0, ≥1.7 mmol/L =1 |
| High-density  lipoprotein cholesterol | HDL-C | <1.0 mmol/L =0, ≥1.0 mmol/L =1 |
| Low-density lipoprotein cholesterol | LDL-C | <3.4 mmol/L =0, ≥3.4 mmol/L =1 |
| Impaired fasting glucose | IFG | Fasting plasma glucose <5.6 mmol/L =0 (No), 5.6-6.9 mmol/L =1 (Yes) |
| New-onset diabetes | Diabetes | No=0, Yes=1 |

Supplementary table 2. The strength of the conditional dependence relationships between nodes.

| from | to | strength |
| --- | --- | --- |
| Sex | Education | -327.526 |
| Sex | Smoking | -2536 |
| Sex | Drinking | -478.439 |
| Sex | WC | -349.49 |
| Sex | Snoring | -84.3484 |
| Sex | WHR | -1169.33 |
| Sex | LDL_C | -141.865 |
| Sex | Daytime_nap | -74.2254 |
| Sex | HDL_C | -75.4357 |
| Sex | HR | -32.2067 |
| Age | Drinking | -340.884 |
| Age | Education | -754.808 |
| Age | Family_income | -117.857 |
| Age | hxchd | -65.9293 |
| Age | Hypertension | -272.808 |
| Age | Occupation | -173.488 |
| Age | Smoking | -104.587 |
| Age | WC | -145.025 |
| Education | Occupation | -1515.55 |
| Education | Smoking | -114.433 |
| Education | Family_income | -381.46 |
| Occupation | Daytime_nap | -43.883 |
| Smoking | BMI | -19.8274 |
| Drinking | HDL_C | -5.62512 |
| Physical_activity | Drinking | -399.913 |
| CHSCTO | SRGH | -1383.86 |
| Hypertension | CHSCTO | -40.148 |
| Hypertension | Diabetes | -61.9691 |
| Hypertension | HR | -33.1399 |
| Hypertension | IFG | -78.5205 |
| Hypertension | rxlipid | -50.0308 |
| rxlipid | TG | -34.746 |
| Family_history | IFG | -2.11135 |
| BMI | Hypertension | -76.3824 |
| BMI | Snoring | -174.433 |
| BMI | WHR | -96.8059 |
| BMI | WC | -2253.3 |
| WC | Hypertension | -39.5649 |
| WC | TG | -129.871 |
| WC | Diabetes | -39.757 |
| WC | WHR | -982.959 |
| WC | IFG | -104.408 |
| WHR | TG | -77.9057 |
| TG | LDL_C | -183.359 |
| TG | HDL_C | -68.686 |
| IFG | Diabetes | -243.501 |

Note.

1. The strength of the conditional dependence relationships was measured using Bayesian information criterion score gain or loss, which would be caused by each arc’s removal. Therefore, negative values correspond to decreases in the network score and positive values correspond to increases in the network score (i.e. the stronger the relationship, the more negative the difference).
2. The nodes in the BN model, as follows: Sex; Age; Education; Occupation; Family income; Smoking; Drinking; Physical activity; Daytime nap=daytime napping; Snoring; CHSCTO=current health status compared to others; SRGN=self-reported general health; Hypertension; HR=heat rate; rxlipid=lipid lowering drugs; hxchd=self-reported coronary heart disease; Family history=family history of diabetes; BMI=body mass index; WC=waist circumference; WHR=waist-to-hip ratio; TG=triglycerides; HDL-C=high-density lipoprotein-cholesterol; LDL-C=low-density lipoprotein cholesterol; IFG=impaired fasting glucose.

Supplementary table 3. Post-test probability table of the deterministic nodes by sex

| Hypertension | IFG | WC, cm | Post-test probability  Diabetes | 95% CI |
| --- | --- | --- | --- | --- |
| Women | | | | |
| No | No | <80 | 0.022 | (0.021-0.022) |
| No | No | ≥80 | 0.047 | (0.446-0.048) |
| Yes | No | <80 | 0.066 | (0.065-0.067) |
| Yes | No | ≥80 | 0.095 | (0.093-0.096) |
| No | Yes | <80 | 0.104 | (0.103-0.106) |
| No | Yes | ≥80 | 0.165 | (0.165-0.169) |
| Yes | Yes | <80 | 0.161 | (0.159-0143） |
| Yes | Yes | ≥80 | 0.287 | (0.283-0.288) |
| Men | | | | |
| No | No | - | 0.022 | (0.021-0.022) |
| Yes | No | - | 0.063 | (0.062-0.064) |
| No | Yes | - | 0.133 | (0.131-0.135) |
| Yes | Yes | - | 0.182 | (0.180-0.184) |

IFG; impaired fasting glucose; WC, waist circumference; CI, confidence interval.

Supplementary table 4. Forward stepwise logistic regression model with 10 risk factors of new-onset diabetes and prediction model performance in all participants

| **Variables** | $\boldsymbol{\beta}$ | ***P*-values** | **Adjusted odds ratio (95% CI)** |
| --- | --- | --- | --- |
| **Age, years** |  |  |  |
| <55 | 0.00 |  | 1.00 |
| 55-64 | 0.27 | 0.009 | 1.31(1.07-1.59) |
| ≥65 | 0.48 | <0.001 | 1.61(1.31-2.00) |
| **Men** | -0.37 | <0.001 | 0.69(0.59-0.81) |
| **Hypertension, yes/no** | 0.58 | <0.001 | 1.79(1.56-2.07) |
| **Heart rate, beats/min** |  |  |  |
| <60 | 0.00 |  | 1.00 |
| 60-99 | 0.23 | 0.17 | 1.26(0.92-1.78) |
| ≥100 | 0.49 | 0.04 | 1.63(1.01-2.63) |
| **Family history of diabetes, yes/no** | 0.55 | <0.001 | 1.74(1.44-2.09) |
| **BMI, kg/m^2^** |  |  |  |
| <25.0 | 0.00 |  | 1.00 |
| 25.0-29.9 | 0.35 | <0.001 | 1.42(1.23-1.64) |
| ≥30.0 | 0.68 | <0.001 | 1.98(1.49-2.61) |
| **WHR ≥0.9, yes/no** | 0.44 | <0.001 | 1.56(1.34-1.81) |
| **TG ≥1.70 mmol/l, yes/no** | 0.55 | <0.001 | 1.73(1.51-1.98) |
| **HDL-C, ≥1.0 mmol/l, yes/no** | -0.41 | 0.02 | 0.66(0.47-0.94) |
| **IFG, yes/no** | 1.40 | <0.001 | 4.05(3.54-4.65) |
| Prediction model performance in validation set | | | |
| Accuracy | 0.573 |  |  |
| Sensitivity | 0.850 |  |  |
| Specificity | 0.548 |  |  |
| AUC | 0.752 (0.723-0.782) | |  |

CI, confidence interval; BMI, body mass index; TG, triglycerides; HDL-C, high-density lipoprotein-cholesterol; TG, triglycerides; IFG, impaired fasting glucose; WHR, waist-to-hip ratio; AUC, area under receiver operating characteristic curve. Prediction model included 10 factors above.

Supplementary table 5. Forward stepwise logistic regression model with 11 risk factors of new-onset diabetes and prediction model performance in women.

| **Variables** | $\boldsymbol{\beta}$ | ***P*-values** | **Adjusted odds ratio (95% CI)** |
| --- | --- | --- | --- |
| **Age, years** |  |  |  |
| <55 | 0.00 |  | 1.00 |
| 55-64 | 0.27 | 0.02 | 1.30(1.05-1.62) |
| ≥65 | 0.42 | <0.001 | 1.52(1.20-1.93) |
| **Smoking** |  |  |  |
| Never smokers | 0.00 |  | 1.00 |
| Former smokers | -0.83 | 0.04 | 0.44(0.18-0.91) |
| Current smokers | -0.08 | 0.82 | 0.93(0.44-1.73) |
| **Physical activity** |  |  |  |
| Inactive | 0.00 |  | 1.00 |
| Moderately active | 0.23 | 0.16 | 1.26(0.92-1.76) |
| Active | -0.03 | 0.86 | 0.97(0.71-1.35) |
| **Hypertension, yes/no** | 0.68 | <0.001 | 1.97(1.67-2.31) |
| **Family history of diabetes, yes/no** | 0.59 | <0.001 | 1.80(1.44-2.23) |
| **BMI, kg/m^2^** |  |  |  |
| <25.0 | 0.00 |  | 1.00 |
| 25.0-29.9 | 0.36 | <0.001 | 1.43(1.18-1.74) |
| ≥30.0 | 0.62 | <0.001 | 1.85(1.31-2.58) |
| **WC,≥80 cm, yes/no** | 0.19 | 0.09 | 1.20(0.97-1.49) |
| **WHR ≥0.9, yes/no** | 0.32 | 0.001 | 1.38(1.13-1.67) |
| **TG ≥1.70 mmol/l, yes/no** | 0.43 | <0.001 | 1.53(1.31-1.80) |
| **HDL-C ≥1.0 mmol/l, yes/no** | -0.61 | 0.02 | 0.55(0.33-0.94) |
| **IFG, yes/no** | 1.36 | <0.001 | 3.90(3.33-4.57) |
| Prediction model performance in validation set | | | |
| Accuracy | 0.658 |  |  |
| Sensitivity | 0.833 |  |  |
| Specificity | 0.642 |  |  |
| AUC | 0.803 (0.774-0.832) | |  |

CI, confidence interval; BMI, body mass index; WC, waist circumference; WHR, waist-to-hip ratio; TG, triglycerides; HDL-C, high-density lipoprotein-cholesterol; IFG, impaired fasting glucose; AUC, area under receiver operating characteristic curve. Prediction model included the 11 factors above.

Supplementary table 6. Forward stepwise logistic regression model with nine risk factors of new-onset diabetes and prediction model performance in men

| **Variables** | | $\boldsymbol{\beta}$ | **P-values** | | **Adjusted odds ratio (95% CI)** | |  |
| --- | --- | --- | --- | --- | --- | --- | --- |
| **Occupation** | |  |  | |  | |  |
| Manual | | 0.00 |  | | 1.00 | |  |
| Non-manual | | 0.31 | 0.03 | | 1.37(1.04-1.80) | |  |
| Others | | -0.02 | 0.92 | | 0.98(0.63-1.48) | |  |
| **Hypertension, yes/no** | | 0.56 | <0.001 | | 1.74(1.32-2.31) | |  |
| **Heart rate, beats/min** | |  |  | |  | |  |
| <60 | | 0.00 |  | |  | |  |
| 60-99 | | -0.25 | 0.33 | | 0.78(0.49-1.32) | |  |
| ≥100 | | 0.64 | 0.08 | | 1.90(0.92-4.12) | |  |
| **Lipid lowering drugs, yes/no** | | 0.65 | 0.01 | | 1.92(1.12-3.19) | |  |
| **Family history of diabetes, yes/no** | | 0.34 | 0.08 | | 1.41(0.94-2.06) | |  |
| **WHR ≥0.9, yes/no** | | 0.56 | <0.001 | | 1.74(1.32-2.31) | |  |
| **TG ≥1.70 mmol/l, yes/no** | | 0.32 | 0.02 | | 1.38(1.05-1.82) | |  |
| **HDL-C ≥1.0 mmol/l, yes/no** | -0.42 | | | 0.09 | | 0.66(0.41-1.09) | |
| **IFG, yes/no** | | 1.51 | < 0.001 | | 4.51(3.45-5.93) | |  |
| Prediction model performance in validation set | | | | | | |  |
| Accuracy | | 0.731 |  | |  | |  |
| Sensitivity | | 0.697 |  | |  | |  |
| Specificity | | 0.734 |  | |  | |  |
| AUC | | 0.741 (0.686-0.796) | | |  | |  |

CI, confidence interval; WHR, waist-to-hip ratio; TG, triglycerides; HDL-C, high-density lipoprotein-cholesterol; IFG, impaired fasting glucose; AUC, area under receiver operating characteristic curve. Prediction model included the 9 factors above.

Supplementary table 7. Logistic regression (enter) model with three factors of new-onset diabetes and prediction model performance in all participants

| **Variables** | $\boldsymbol{\beta}$ | **P-values** | **Adjusted odds ratio (95% CI)** |
| --- | --- | --- | --- |
| **Hypertension, yes/no** | 0.71 | <0.001 | 2.03(1.78-2.33) |
| **WC, ≥90/80 cm in men or ≥80 cm in women, yes/no** | 0.61 | <0.001 | 1.84(1.61-2.10) |
| **IFG, yes/no** | 1.46 | <0.001 | 4.32(3.77-4.94) |
| Prediction model performance in validation set | | | |
| Accuracy | 0.734 |  |  |
| Sensitivity | 0.604 |  |  |
| Specificity | 0.745 |  |  |
| AUC | 0.734, 95% CI (0.703-0.764) | |  |

CI, confidence interval; WC, waist circumference; IFG, impaired fasting glucose; AUC, area under receiver operating characteristic curve. Prediction model included 3 factors above.

**Code**

We provide code for the analysis in this paper.

1 Code for loading required packages:

library(tidyverse)

library(haven)

library(expss)

library(MASS)

library(bnlearn)

library(Rgraphviz)

library(reportROC)

library(mice)

library(readxl)

library(writexl)

library(pROC)

library(car)

library(caret)

library(caTools)

2 Code for BN model

ensemble <- function(data, bootstrapn, bootstrapt) {

      bl=  matrix(c( ),

  ncol = 2, byrow = TRUE, dimnames = list(NULL, c("from", "to"))

    )

wl <- matrix(c( ),

ncol = 2, byrow = TRUE, dimnames = list(NULL, c("from", "to")))

    output = data.frame(from=c(), to=c())

    for(seed in 1:bootstrapn){

       set.seed(seed)

      newdata = data[sample(1:nrow(data), nrow(data), replace=T),]

      fit <- structural.em(newdata, maximize = "hc", maximize.args = list(blacklist = bl), fit = "bayes",

      fit.args = list(), impute = "parents", impute.args = list(), return.all = FALSE,

      start = NULL, max.iter = 100, debug = FALSE)

      arcs = as.data.frame(fit$arcs)

      output = rbind(output, arcs)

    }

    output$arc = paste0(output$from, output$to)

    fre = as.data.frame(table(output$arc))

    fre=fre[which(fre$Freq>=bootstrapn*bootstrapt),]

    output6=unique(merge(output, fre, by.x="arc",by.y="Var1"))

    return (output6)

}

3 Code for AUC

set.seed( )

five_folds <- createFolds(data , k = 5, list = TRUE, returnTrain = FALSE)

auc_list = list()

dag_list = list()

train_y = NULL

train_pred = NULL

test_y = NULL

test_pred = NULL

d = NULL

for (i in five_folds) {

    set.seed( )

    data = as.data.frame(data_all[-i,])

    r_model <-  bn.fit(r, data,method = "bayes")

    data = impute(r_model, data)

    pre_train <- predict(r_model, node="Diabetes", data, method = "bayes-lw", prob = TRUE)

    x = attr(pre_train, "prob")[2,]

    fold_data_test = as.data.frame(data_all[i,])

    data = impute(r_model, data)

    data <- predict(r_model, node="Diabetes", data, method="bayes-lw", prob = TRUE)

    fold_pred = attr(data, "prob")[2,]

    ttt <- reportROC(gold= data $Diabetes, predictor = fold_pred, positive = "l", plot = FALSE)

    auc_list[[length(auc_list) + 1]] <- ttt

    train_y = c(train_y, data$iabetes)

    train_pred = c(train_pred, x)

    test_y <- c(test_y, Diabetes)

    test_pred <- c(test_pred, data)

}

4 Code for diagrams

ggplot(data, mapping=aes(x = x2,y = y, fill = ,pattern= , pattern_type= ), size = 20) +

geom_bar_pattern(stat = "identity", position = "dodge", pattern_fill = "black",

fill = "white", colour = "black", pattern_spacing = 0.01,

pattern_frequency = 5, pattern_angle = 45) +

ggpubr::theme_pubr() +

theme(legend.position = "right") +

theme( panel.background = element_rect(colour = "black", size=0.5)) +

labs(x = "", y = "Post-test probability")+

scale_y_continuous(breaks=seq(0, 0.2, 0.05), limits = c(0, 0.2)) +

scale_pattern_manual(values=c('stripe', 'none')) +

scale_pattern_type_manual(values=c(NA, NA))

scale_fill_hue(name=" ")

5 Code for logistic model

formatFit<-function(fit){

p<-summary(fit)$coefficients[,4]

wald<-summary(fit)$coefficients[,3]^2

valueB<-coef(fit)

valueOR<-exp(coef(fit))

confitOR<-exp(confint(fit))

data.frame(

B=round(valueB,3),

Wald=round(wald,3),

OR_with_CI=paste(round(valueOR,3),"(",

round(confitOR[,1],3),"~",round(confitOR[,2],3),")",sep=""),

P=format.pval(p,digits = 3,eps=0.001)

)

}

p<-glm(Diabetes ~ . ,family=binomial, data=l_train)

step.model <- stepAIC(p)

summary(step.model)

vif(step.model, digits = 3)

formatFit(step.model)
